# GED-0507 attenuates lung fibrosis by counteracting myofibroblast transdifferentiation *in vivo* and *in vitro*

**DOI:** 10.1371/journal.pone.0257281

**Published:** 2021-09-16

**Authors:** Silvia Speca, Caroline Dubuquoy, Christel Rousseaux, Philippe Chavatte, Pierre Desreumaux, Paolo Spagnolo

**Affiliations:** 1 Univ. Lille, INSERM, U1286 –Infinite–Institute for Translational Research in Inflammation, Lille, France; 2 Intestinal Biotech Development, Lille, France; 3 Laboratoire de Pharmacie Clinique, Faculté des Sciences Pharmaceutiques et Biologiques, Lille, France; 4 Hepato-Gastroenterology Department, CHU Lille, Lille, France; 5 Respiratory Disease Unit, Department of Cardiac, Thoracic, Vascular Sciences and Public Health, University of Padova, Padova, Italy; Virginia Commonwealth University Medical Center, UNITED STATES

## Abstract

The development of more effective, better tolerated drug treatments for progressive pulmonary fibrosis (of which idiopathic pulmonary fibrosis is the most common and severe form) is a research priority. The peroxisome proliferator-activated receptor gamma (PPAR-γ) is a key regulator of inflammation and fibrosis and therefore represents a potential therapeutic target. However, the use of synthetic PPAR-γ agonists may be limited by their potentially severe adverse effects. In a mouse model of bleomycin (BLM)-induced pulmonary fibrosis, we have demonstrated that the non-racemic selective PPAR-γ modulator GED-0507 is able to reduce body weight loss, ameliorate clinical and histological features of pulmonary fibrosis, and increase survival rate without any safety concerns. Here, we focused on the biomolecular effects of GED-0507 on various inflammatory/fibrotic pathways. We demonstrated that preventive and therapeutic administration of GED-0507 reduced the BLM-induced mRNA expression of several markers of fibrosis, including transforming growth factor (TGF)-β, alpha-smooth muscle actin, collagen and fibronectin as well as epithelial-to-mesenchymal transition (EMT) and expression of mucin 5B. The beneficial effect of GED-0507 on pulmonary fibrosis was confirmed *in vitro* by its ability to control TGFβ-induced myofibroblast activation in the A549 human alveolar epithelial cell line, the MRC-5 lung fibroblast line, and primary human lung fibroblasts. Compared with the US Food and Drug Administration-approved antifibrotic drugs pirfenidone and nintedanib, GED-0507 displayed greater antifibrotic activity by controlling alveolar epithelial cell dysfunction, EMT, and extracellular matrix remodeling. In conclusion, GED-0507 demonstrated potent antifibrotic properties and might be a promising drug candidate for the treatment of pulmonary fibrosis.

## Introduction

Peroxisome proliferator-activated receptor-gamma (PPAR-γ) is a ligand-inducible nuclear transcription receptor that has a key role in organ homeostasis and downregulates inflammation and fibrosis [1–3]. Thanks to its broad regulatory activities and ability to bind a variety of natural or synthetic ligands, PPAR-γ is a potential therapeutic target in a range of inflammatory and fibrotic diseases [2, 4]. However, the majority of synthetic full PPAR-γ agonists are associated with serious adverse effects—possibly due to both their stereochemistry and their potential conversion into various metabolically active intermediates. Computational-chemistry-based studies have driven the development of highly efficacious small chiral nonracemic ligands that differentially activate PPAR-γ without inducing adverse effects. These compounds are referred to as selective PPAR modulators (SPPARMs).

The SPPARM (S)-(−)-GED-0507-34 (GED-0507) was developed as 5-aminosalycilate analog but has stronger PPARγ-mediated anti-inflammatory activity and a good safety/tolerability profile [4].

We have shown previously that GED-0507 exerts antifibrotic activity in a mouse model of bleomycin (BLM)-induced pulmonary fibrosis and in a mouse model of experimental dextran sodium sulfate (DSS)-induced intestinal inflammation [3]. Specifically, we demonstrated that GED-0507 reduced intestinal fibrosis in the DSS mouse model by inhibiting the TGFβ/Smad pathway, which is activated by persistent tissue injury and chronic inflammation. Consequently, several cytokines, growth factors and chemokines are produced in an uncontrolled manner, leading to the activation of myofibroblasts—a profibrotic cellular phenotype that expresses alpha-smooth muscle actin (αSMA). Myofibroblasts are derived from the transdifferentiation of several cell types, (including fibroblasts, epithelial cells, endothelial cells, and vascular smooth muscle cells) and synthesize large amounts of extracellular matrix (ECM) components. Excessive ECM deposition leads to parenchymal distortion and loss of function of the affected organ, and is a feature of all fibrotic diseases [5].

Pulmonary fibrosis is a frequent complication of interstitial lung disease (ILD), a large and heterogeneous group of conditions characterized by various degrees of inflammation and fibrosis [6]. Idiopathic pulmonary fibrosis (IPF) is the most common and severe form of ILD, with a median survival time from diagnosis is 3 to 5 years if untreated [7, 8]. According to current estimates, approximately 3 million people worldwide are affected by IPF. The 2015 guidelines on the treatment of IPF posed a conditional (i.e., weak) recommendation for the use of two drugs: pirfenidone (Pirf), a compound with anti-inflammatory, antioxidant and antifibrotic properties and nintedanib (Nint), an intracellular inhibitor of triple tyrosine kinases receptors, such as the vascular endothelial growth factor receptor 1–3, the fibroblast growth factor receptor 1–3, and the platelet-derived growth factor receptor a and b [9]. Pivotal clinical trials, systematic reviews and meta-analyses indicate for both drugs similar efficacy in slowing down functional decline and disease progression in IPF [10–12]. However, neither drug improves or even stabilizes lung function, and both have tolerability issues and significant discontinuation rates [13]. As such, there is high unmet need for more efficacious and better tolerated therapeutics [14].

The objectives of the present study were to characterize *in vivo* the biomolecular effects of GED-0507 in a murine model of BLM-induced pulmonary fibrosis and to evaluate *in vitro* its ability to prevent immortalized human epithelial cell and fibroblast lines and primary human lung fibroblasts (HLFs) from differentiating towards a pro-fibrotic profile [15–18].

## Materials and methods

### Animals and ethics statement

Eight-week-old C57BL/6 mice (n = 108) were purchased from Janvier Labs (Le Genest-Saint-Isle, France) and maintained in an accredited pathogen-free facility at the Pasteur Institute of Lille (Lille, France). Mice were provided with standard chow and ad libitum access to water. The mice were randomized to treatment with (A) PBS (n = 12), BLM (n = 22), BLM+GED-0507 (n = 20), and BLM+Pirf (n = 10) with preventive intent, and (B) PBS (n = 5), BLM (n = 10), BLM+ GED-0507 (n = 10), BLM+Nint (n = 10) and BLM+Pirf (n = 9) with curative intent. In order to ensure adequate statistical power, the sample size for each group was defined by considering the previously reported mortality rate induced by BLM administration [7]. Distress and body weight loss are clinical endpoints in the murine model of BLM-induced lung fibrosis, and so supportive care for minimizing these effects was not provided. However, the mice were observed daily for signs of distress and monitored weekly for body weight loss. All experiments were carried out in accordance with the European Directive No. 2010/63/EU, revising Council Directive No. 86/609/EEC of November 24, 1986 (number of ethic protocol: 2020061914271271). Animal handling and euthanasia were carried out by qualified personal for animal experimentation, in accordance with the French directive No. 2013–118 of 1st February 2013.

### Compounds

BLM sulfate was purchased from Sigma Aldrich (Saint-Quentin-Fallavier, France), GED-0507 was purchased from PPM Services SA (Morbio Inferiore, Switzerland), Pirf was bought from TCI Europe N.V. (Boerenveldseweg, Belgium), and Nint came from Interchim (Kampenhout, Belgium).

### Experimental design in mice

Lung fibrosis was induced in mice by a single intratracheal instillation of 0.8 mg/kg (0.025 U/kg) BLM. Starting on day 1 after BLM administration (for treatment with preventive intent) or day 14 (for treatment curative intent), mice were treated daily by oral gavage with optimal concentrations of GED-0507 (100 mg/Kg/day), Pirf (400 mg/Kg/day) or Nint (60 mg/Kg/day) dissolved in 0.5% carboxymethylcellulose + 1% v/v Tween 80. The direct effects of each compound tested were observed 28 days after the BLM administration. The study design is summarized in S1 Fig.

After excision, left lungs were fixed in 4% buffered formaldehyde and embedded in paraffin for histological analysis, whereas right lungs were dissected and stored at -80°C for molecular analysis.

### A549 and MRC-5 cell lines and primary HLFs

A549 (ATCC® CCL-185™, Molsheim Cedex, France) and MRC-5 (ATCC® CCL171™) cell lines were grown in Dulbecco’s modified Eagle’s medium supplemented with 100 U/ml penicillin, 100 μg/ml streptomycin and 10% fetal bovine serum (FBS). HLFs (ATCC® PCS-201-013™, *Homo Sapiens*, Normal Lung) were grown in Fibroblast Basal Medium (ATCC® PCS-201-030™) supplemented with 100 U/ml penicillin, 100 μg/ml streptomycin and a Fibroblast Growth Kit-Low serum (ATCC® PCS-201-041™), according to the manufacturer’s instructions. Cells were maintained in a humidified atmosphere with 95% air and 5% CO_2_ at 37°C. For each cell type, myofibroblast differentiation was obtained by incubation with human recombinant TGFβ1 (T7039, Sigma Aldrich, Saint-Quentin-Fallavier, France) at optimal concentrations of 20 ng/ml for A549 cells, 10 ng/mL for MRC-5 cells, and 5 ng/mL for HLFs.

### The cell viability assay

Cell viability was measured using an MTT assay (CellTiter 96® Non-Radioactive Cell Proliferation Assay, Promega, Charbonnieres Les Bains, France), according to the manufacturer’s instructions. A549 cells, MRC-5 cells and HLFs were exposed to growing concentrations of GED-0507 (0.1, 1, 10, and 30 mM), Pirf (0.1, 0.4, 0.8, and 1.6 mM) and Nint (0.1, 0.5, 1, and 10 μM) over 2, 4 or 6 days. Pirf and Nint were dissolved in dimethyl sulfoxide (DMSO) (D4540, Sigma Aldrich), ensuring that the concentration of DMSO in the treatment medium did not exceed 1% v/v. The optimal *in vitro* doses of GED-0507, Pirf and Nint were selected after time- and dose-dependent investigations in each cell line, in order to modulate the main fibrotic pathways without reducing cell viability [2, 19, 20].

### Picrosirius red staining

Collagen deposition was determined by picrosirius red staining of paraffin-embedded lung sections or cultured cells. Briefly, 4 μm dried tissue sections or monolayers of 2%-paraformaldehyde (PFA)-fixed A549 and MRC-5 cells were stained with 0.1% direct red stain (365548, Sigma Aldrich)/0.5% picric acid (36011, Sigma Aldrich) for 1 h at room temperature (picrosirius red staining) [21]. Each slide was examined and analyzed remotely using Image J software (W.S. Rasband, National Institutes of Health, Bethesda, MD; http://rsb.info.nih.gov/ij/, 1997–2011), and the mean staining intensity was quantified with the threshold detection method on grayscale images taken from three different microscope fields (magnification: x20).

For cell monolayers, bound picrosirius red was eluted by incubation for 1 h at 37°C with 0.1 N NaOH. Solubilized dye was recovered after each treatment and the absorbance was measured at 550 nm. The results were normalized against the cell density in the remaining monolayers after staining with the DNA-binding dye Crystal Violet for 30 min at room temperature. After elution with pure methanol, the dye’s absorbance at 540 nm was measured [22].

### α-SMA immunofluorescent staining

α-SMA protein expression on 4-μm fixed lung sections and in cell lines (A549 and MRC-5 cells) was evaluated using an immunofluorescence assay. Briefly, after permeabilization in 0.1% Triton-X 100, lung sections and cells were incubated overnight at 4°C with a polyclonal anti-α-SMA primary antibody (ab5694, Abcam, Paris, France) at the dilution of 1:200. Subsequently, the samples were incubated with a specific conjugated anti-mouse secondary antibody AlexaFluor 488 (A32723, ThermoFisher Scientific, Illkirch Cedex, France) for 1 h at room temperature. Nuclei were stained with Hoechst 33342 dye (62249, ThermoFisher Scientific). The staining was quantified using Image J software and the threshold detection method on grayscale images taken from three different microscope fields (magnification: x20/x40) [23]. Data were expressed as fold change in the mean ± standard error of the mean (SEM) staining intensity/total area for the treatment group vs. the control group.

### Quantitative RT-PCRs

Gene expression levels of pro-fibrotic markers (such as TGFβ, α-SMA, collagen, and fibronectin) were measured in lung tissues, cell lines, and HLFs using quantitative real time polymerase chain reactions (qPCRs). The mRNA levels of pro-inflammatory cytokines (such as the interleukin (IL)-1β and the tumor necrosis factor (TNF)-α) epithelial cell markers (E-cadherin and occludin) and mucins (MUC5B) were also measured. Briefly, mRNA was extracted using an RNA purification kit from Macherey-Nagel (Hœrdt, France) and reverse-transcribed to cDNA. The qPCRs were run on a Light Cycler FastStart DNA Master SYBR Green I system (4385617, ThermoFisher Scientific), according to the manufacturer’s instructions. The primers’ sequences and NCBI references are listed in S1 and S2 Tables. Relative gene expression was calculated as E = 2^-ΔCt^, where ΔCt is the difference between the critical threshold cycle (Ct) values of each gene and the relative Ct of GAPDH. Data were expressed as the fold change in the mean ± SEM relative gene expression value for the treatment group vs. the control group.

### Western blotting

Protein expression levels of α-SMA, collagen and fibronectin were determined by Western blotting of HLF extracts. Briefly, total proteins were extracted using RIPA buffer (R0278, Sigma Aldrich, Saint-Quentin-Fallavier, France) supplemented with 100 mM sodium fluoride, 2 mM sodium orthovanadate, 2 mM sodium pyrophosphate, 1 mM phenylmethylsulphonyl fluoride (Sigma Aldrich) and a standard protease inhibitor cocktail (11836170001, Sigma Aldrich). 50 μg of total protein from each sample were separated by SDS-PAGE electrophoresis using Novex Tris-glycine gels with polyacrylamide gradients ranging from 4 to 12% (Novex™ 4–12% Tris-Glycine Mini Gels, 15-well WedgeWell™, ThermoFisher Scientific) and then transferred onto nitrocellulose membranes using the iBlot 2 Dry Blotting System (ThermoFisher Scientific). The membranes were immunoblotted with specific monoclonal antibodies against α-SMA (67735, Proteintech, Manchester, United Kingdom) at dilution of 1:1000 (overnight at 4°C), collagen (66761, Proteintech) at dilution of 1:1000 overnight at 4°C), fibronectin (66042, Proteintech) at dilution of 1:1000) or β-actin (A5441, Sigma Aldrich) at dilution of 1:10000 (2 h at room temperature). Next, blots were incubated with a secondary anti-rabbit horseradish-peroxidase-conjugated antibody (A6154, Sigma Aldrich) at dilution of 1:10000 for 1 h at room temperature and revealed with chemiluminescent substrate (Immobilon ECL Ultra Western HRP Substrate, Sigma Aldrich), according to the manufacturer’s instructions. The membranes were exposed to autoradiography films (Hyperfilm ECL, ThermoFisher Scientific), and the optical density of target bands was determined using a computer-assisted densitometer and ImageJ software. Protein levels were expressed in optical density units per quantity of total protein. The values for each protein were normalized against the internal control (β-actin) in each sample.

### Statistical analysis

Groups were compared using a Kruskal-Wallis non-parametric analysis of variance. Post-hoc comparisons of pairs of groups were performed using the Wilcoxon rank sum test. Data were expressed as the mean ± SEM. The threshold for statistical significance was set to p<0.05.

## Results

### *In vivo* experiments

#### mRNA expression levels of pro-inflammatory cytokines

**Fig 1:**
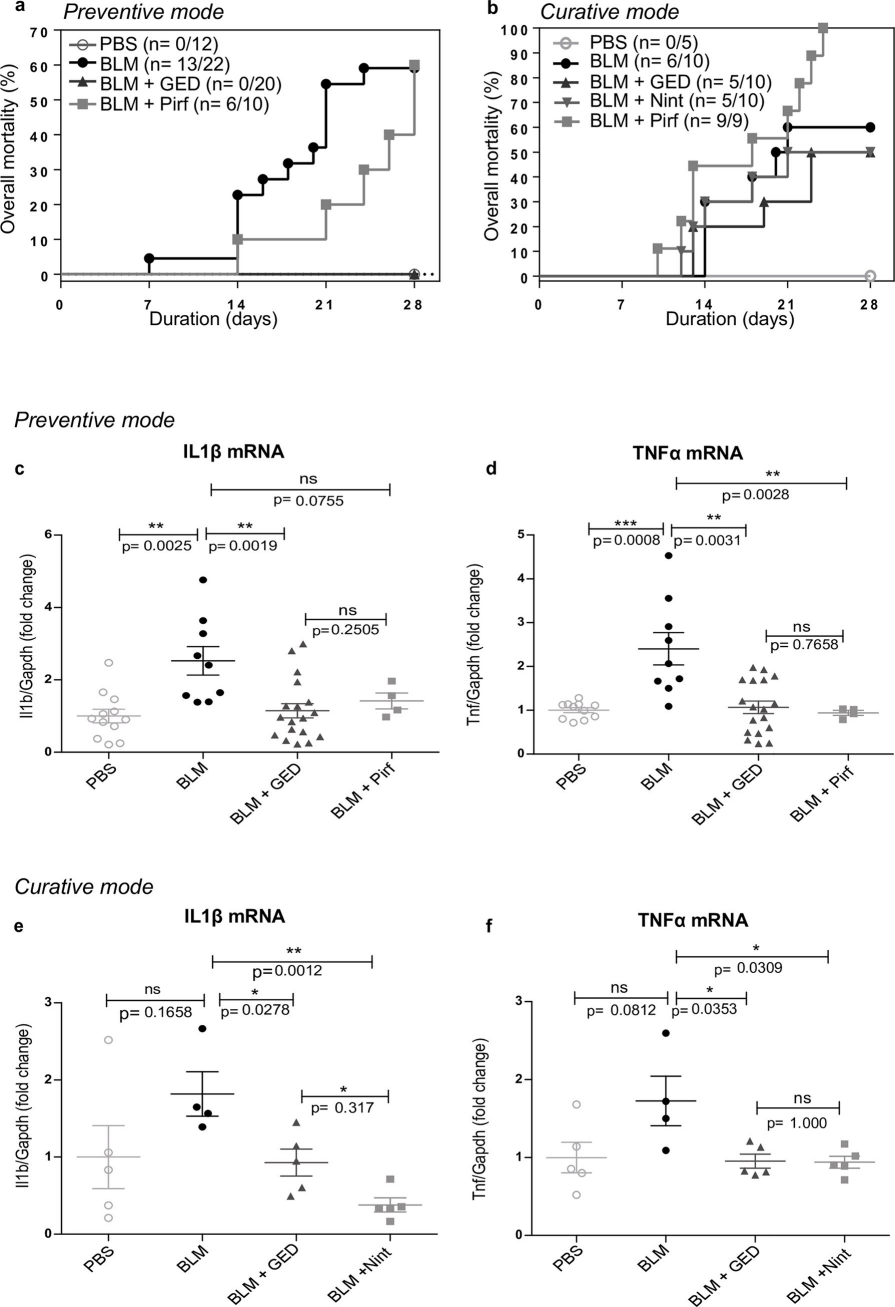
Pro-inflammatory cytokines following the administration of GED-0507, Pirf or Nint with preventive or curative intent. The original groups sizes in the preventative study were n = 12 for PBS, n = 22 for BLM, n = 20 for BLM+GED and n = 10 for BLM+Pirf groups. In the curative study mice were randomized to: PBS (n = 5), BLM (n = 10), BLM+GED (n = 10), BLM+Nint (n = 10) and BLM+Pirf (n = 9). (a,b) Kaplan-Meier curves depicting percent mortality versus time post-BLM. Consistent with our previous report, BLM administration causes 60% mortality rate (13/22 in the preventive study and 6/10 in the curative study). (a) When administered with preventive intent, 100% of GED-receiving mice survives, whereas Pirf treatment is associated to 60% mortality rate. (b) Curative treatments with GED and Nint cause 50% mortality rate (n = 5/10), whereas Pirf administration is associated with 100% mortality at day 24. All survivors are analyzed and reported here. (c-f) Dot plots depicting mRNA expression levels of pro-inflammatory cytokines, (c, e) *Tnf* and (d, f) *Il1b*, measured using quantitative RT-PCR, in frozen lungs of surviving mice after drug administration with preventive or curative intent. The data are quoted as the mean ± SEM fold change vs. PBS-treated mice. * = p< 0.05; ** = p< 0.01; *** = p< 0.001; ns = not significant.

One of the main consequences of BLM treatment is the activation of an early inflammatory response, which is maintained over time. We therefore initially evaluated the mRNA expression levels of pro-inflammatory cytokines. Firstly, we found that the lung expression levels of *Tnf* (the gene encoding TNF-α) and *Il1b* (the gene encoding IL-1β) were higher in BLM-treated mice than in control animals (Fig 1). The preventive administration of GED-0507 and Pirf downregulated the pro-inflammatory gene profile in BLM-treated mice (Fig 1C and 1D). When administered on a curative basis, both GED-0507 and Nint significantly decreased the transcriptional upregulation of *Tnf* and *Il1b* induced by BLM (Fig 1E and 1F). In line with previous observations, curatively administered Pirf resulted in a mortality rate of 100% at day 24, making any further evaluation of the mechanisms and molecular parameters of lung fibrosis unfeasible (Fig 1B) [24].

#### Evaluation of mRNA and protein levels of lung fibrosis markers

In preliminary experiments, we assessed the ability of GED-0507 to reduce weight loss, mortality rate, lung macroscopic lesions, and histological parameters in BLM-treated mice [25]. Here, we performed a more detailed analysis of biomolecular mechanisms related to GED-0507 capacity to control lung fibrosis.

**Fig 2:**
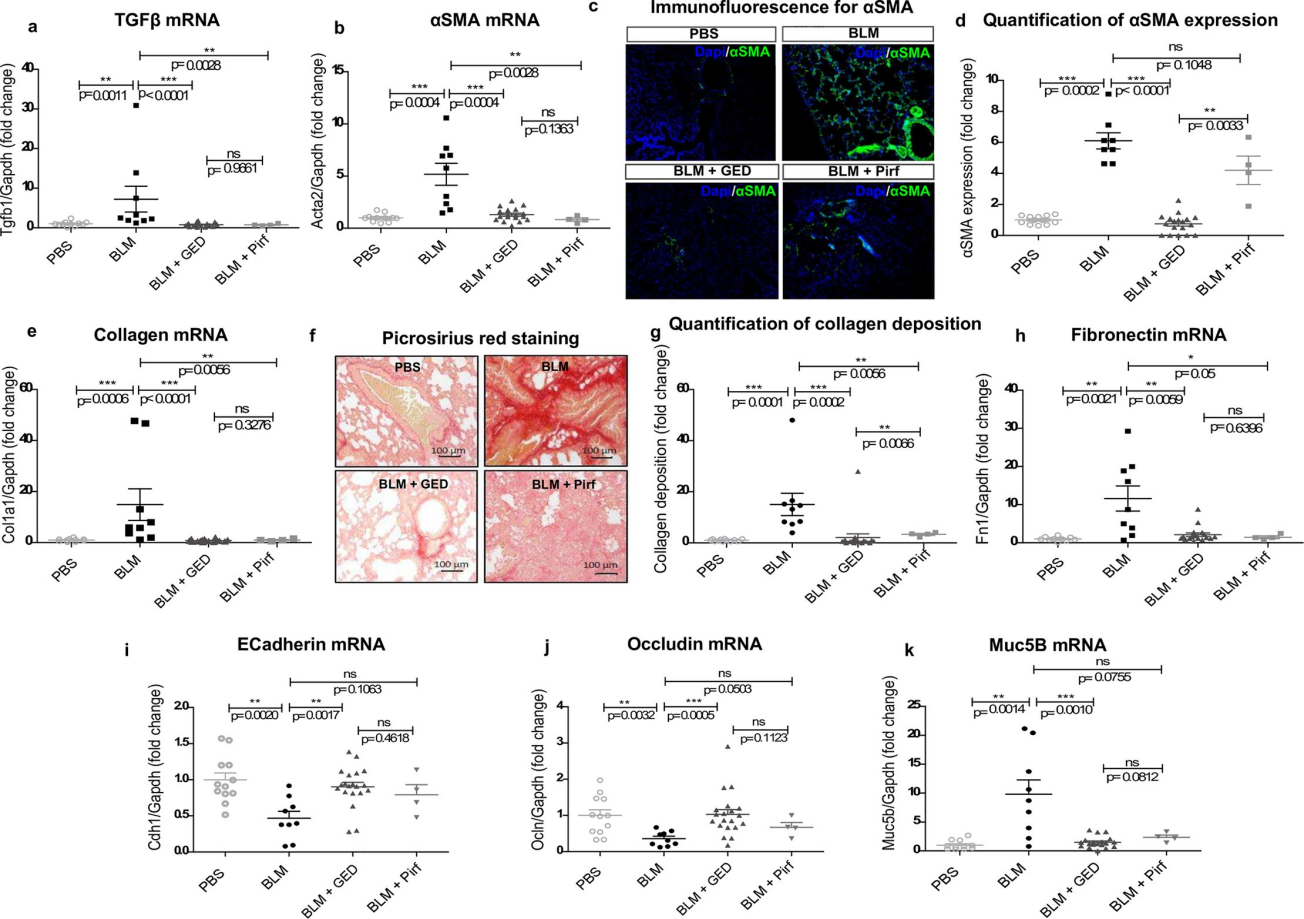
Biomolecular parameters following the administration of GED-0507, Pirf or Nint with preventive intent. For explanation of group size, refer to Fig 1. (a, b, e and h) The dot plots depict mRNA expression levels of *Tgfb*, *Acta2*, *Col1A1* and *Fn1* in frozen lung, as quantified in RT-PCRs. (c) Microphotographs of immunofluorescence staining of α-SMA (green), at a magnification of 40x. Nuclei were counterstained using DAPI (blue). Digital images were processed with the Zeiss LSM Browser, and α-SMA protein expression was quantified on three microscope fields, using the threshold detection method. (d) Scatter plot of α-SMA quantification. (f) Microphotographs of picrosirius red staining on lung sections, for collagen fibers. Images were scanned at a magnification of 40x, and collagen deposition (red) was quantified on digitized images and (g) plotted. (i, j) The loss of epithelial cell markers reflects the pro-fibrotic EMT. Scatter plots show the mRNA expression of epithelial cell markers *Cdh1* and *Ocln*, quantified in RT-PCRs on frozen lungs of all surviving mice. (k) Dot plots depicting mRNA expression levels of *Muc5b*. Data are quoted as the mean ± SEM fold change vs. PBS-treated mice; * = p< 0.05; ** = p< 0.01; *** = p< 0.001; ns = not significant.

**Fig 3:**
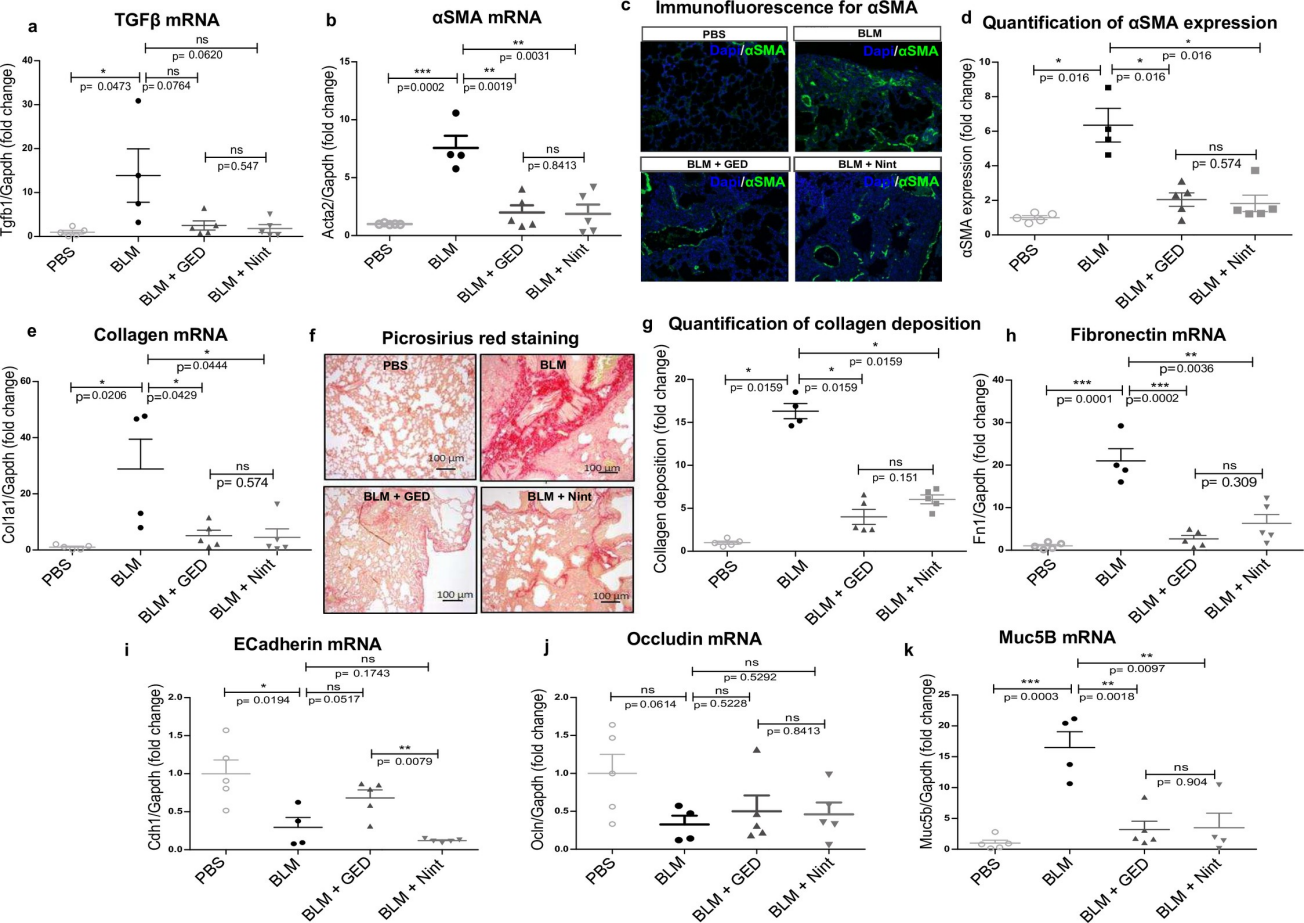
Biomolecular parameters following the administration of GED-0507 and Nint with curative intent. For explanation of group size, refer to Fig 1. (a, b, e and h) Dot plots depicting mRNA expression levels of *Tgfb*, *Acta2*, *Col1A1* and *Fn1* genes quantified in frozen lung in RT-PCRs. (c) Microphotographs of immunofluorescence staining of α-SMA (green) scanned at a magnification of 40x. Nuclei were counterstained using DAPI (blue). Digital images were processed with the Zeiss LSM Browser, and α-SMA protein expression was quantified on three microscope fields, using the threshold detection method. (d) Scatter plot for α-SMA quantification. (f) Microphotographs of picrosirius red staining of lung sections, for collagen fibers. Images were scanned at a magnification of 40x, and collagen deposition (red) was quantified on digitized images and (g) plotted. (i, j) The loss of epithelial cell markers reflects the pro-fibrotic EMT. Scatter plots show the mRNA expression of epithelial cell markers *Cdh1* and *Ocln*, quantified in RT-PCRs on frozen lungs of all surviving mice. (k) Dot plots depicting mRNA expression levels of *Muc5b*. Data are quoted as the mean ± SEM fold change vs. PBS-treated mice. * = p< 0.05; ** = p< 0.01; *** = p< 0.001; ns = not significant.

The pulmonary expression of all tested genes—*Tgfb1* (coding for TGFβ), *Acta2 (*coding for α-SMA), *Col1a1* (coding for collagen) and *Fn1* (coding for fibronectin)—was greater in the BLM-treated mice than in control animals (Figs 2 and 3). Preventive administration of GED-0507 significantly downregulated the BLM-induced mRNA expression of all these markers, with Pirf exerting a similar but less pronounced effect (Fig 2A, 2B, 2E and 2H). GED-0507 also exerted an inhibitory effect on the protein expression of the myofibroblast differentiation marker α-SMA, as observed and quantified in an immunofluorescence assay (Fig 2C and 2D). Protein expression levels of collagen were also lower after treatment with GED-0507, as observed via picrosirius red staining and its relative quantification with digital imaging. Conversely, relative to control BLM-treated animals, preventive treatment with Pirf significantly decreased collagen deposition only (Fig 2F and 2G). Along with the elevated expression of major fibrosis markers, we also observed modulation of the BLM-induced pulmonary epithelial signature. Indeed, the lung tissue expression of genes coding for epithelial cell markers (such as *Cdh1* coding for E-cadherin and *Ocln* coding for occludin) was significantly lower in BLM mice than in control animals. Preventive treatment with GED-0507 restored the loss of E-cadherin and occludin to levels similar to those measured in the lungs of PBS-treated mice. Conversely, Pirf did not prevent the BLM-induced decrease of either E-cadherin or occludin mRNA levels (Fig 2I and 2J). We also found that mRNA expression of MUC5B (encoded by *Muc5b)* in the lung was significantly greater in BLM-treated mice than in control animals. Preventive treatment with GED-0507 was associated with a significantly lower level of transcriptional expression of *Muc5b* in BLM-treated mice, whereas no significant effect was observed in mice receiving Pirf (Fig 2K).

When GED-0507 and Nint were given with curative intent (Fig 3), both compounds were not able to reduce the *Tgfb1* expression levels, nevertheless GED-0507 and Nint were associated with significantly and similarly low levels of *Acta2*, *Col1a1*, *Fn1* transcription (Fig 3A, 3B, 3E and 3H). This transcriptional effect translated into significantly lower levels of protein expression of α-SMA and collagen in mice with lung fibrosis receiving GED-0507 or Nint with curative intent, relative to untreated animals (Fig 3C, 3D, 3F and 3G). In this context, GED-0507 (but not Nint) also attenuated the BLM-induced decrease in *Cdh1* expression, whereas no effect was observed with regard to *Ocln* (Fig 3I and 3J). Compared to BLM-treated mice, gene expression levels of *Muc5B* were also significantly lower following administration of GED-0507 or Nint with curative intent (Fig 3K).

### *In vitro* experiments

#### Cell viability

In order to determine *in vitro* toxicity, we exposed three types of cell to GED-0507, Pirf and Nint. For GED-0507, the results of the cell toxicity assay showed that none of the doses used in the present experiments were cytotoxic after 2, 4 and 6 days of exposure. Conversely, cytotoxic effects were observed in A549 and MRC-5 cell lines treated with the highest concentration of Nint (i.e., 10 μM) and Pirf (i.e., 1.6 μM) on day 2 and day 4, respectively. Based on our time- and dose-dependent assessments of the viability of MRC-5 and A549 cells over two and four days, respectively, the optimal doses were found to be 1–30 mM in 2% FBS culture medium for GED-0507, 0.1–1 μM in 0.2% FBS culture medium + 0.01% DMSO for Nint, and 0.1–0.8 mM + 0.01% DMSO for Pirf. In terms of viability, HLFs were more sensitive to Pirf and Nint than MRC-5 and A549 cells were. Indeed, a cytotoxic effect was observed after 2 days of incubation with Pirf and Nint at doses of 0.8 mM and 1 μM, respectively. The highest tolerated non-cytotoxic concentrations were 0.4 mM and 0.5 μM for Pirf and Nint, respectively, after a maximum stimulation time of 2 days. Therefore, a study design including two days of incubation with GED-0507 (1–30 mM), Nint (0.1–0.5 μM) and Pirf (0.1–0.4 mM) was applied to HLFs (S2 Fig).

#### Evaluation of the ability of GED, Nint and Pirf to counter TGFβ-induced myofibroblast activation in the MRC-5 lung fibroblast cell line

**Fig 4:**
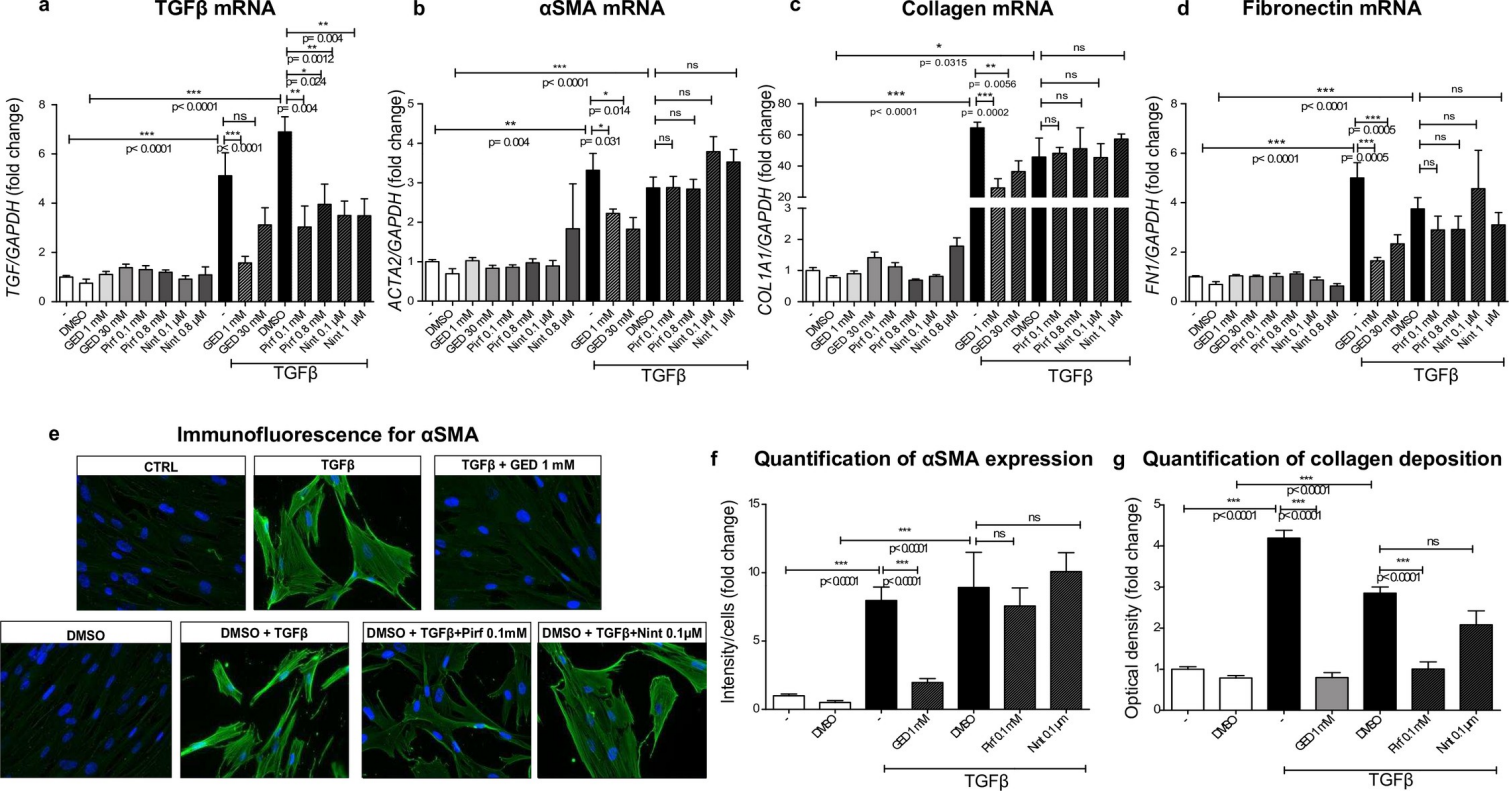
*In vitro* evaluation of myofibroblast activation in MRC-5. Myofibroblast activation was induced in MRC-5 human fibroblasts stimulated with 10 ng/mL TGFβ over two days. GED-0507 (1–30 mM), Pirf (0.1–0.8 mM) or Nint (0.1–1 μM) were administered at the same time as TGFβ. mRNA expression levels for (a) *TGFB1*, (b) *ACTA*, (c) *COL1A1* and (d) *FN1* were quantified in RT-PCRs and converted into histograms for three independent experiments performed in triplicate. (e) Microphotographs of immunofluorescence staining of α-SMA (green) were scanned at a magnification of 40x. Nuclei were counterstained using DAPI (blue). Digital images were processed with the Zeiss LSM Browser and (f) α-SMA protein expression was quantified on 3 microscope fields, using the threshold detection method with Image J software and represented on relative scatter plots. (g) Collagen deposition was quantified by picrosirius red staining and converted into histograms for 3 independent experiments performed in triplicate. Since Pirf and Nint were solubilized in DMSO, DMSO- and/or TGFβ+DMSO-treated cells were used as comparators in the statistical analysis. Data are quoted as the mean ± SEM fold change vs. CTRL cells. * = p< 0.05; ** = p< 0.01; *** = p< 0.001.

The fibroblast-to-myofibroblast transition in MRC-5 cells was induced by 10 ng/mL TGFβ stimulation over two days, leading to a significant elevation in expression of the main fibrosis markers at both the mRNA and protein levels (Fig 4). At a concentration of 1 mM, GED-0507 was able to counter the TGFβ-induced upregulation of *TGFB1* expression. The TGFβ-induced expression of all the other tested pro-fibrotic genes (such as *ACTA2*, *COL1A1*, and *FN1*) was equally reduced by both doses of GED-0507 (1 and 30 mM). Conversely, the expression of these genes was not significantly modulated when MRC-5 cells were treated with Pirf or Nint under the same conditions (Fig 4A–4D).

Furthermore, GED-0507 was able to exert an inhibitory effect on α-SMA and collagen protein expression, as observed and quantified in an immunofluorescence assay and picrosirius red staining, respectively (Fig 4E and 4G). Pirf was able to reduce TGFβ-induced collagen deposition but not the α-SMA protein expression (Fig 4G). The discrepancy between the mRNA and protein expression levels for collagen in Pirf-treated cells is consistent with the fact that the mRNA expression is related specifically to collagen I (the *COL1A1* gene) whereas picrosirius red stains all subtypes of collagens deposed by the cell.

#### Evaluation of the ability of GED, Nint and Pirf to counter the TGFβ-induced mesenchymal transition in alveolar epithelial cells (A549)

**Fig 5:**
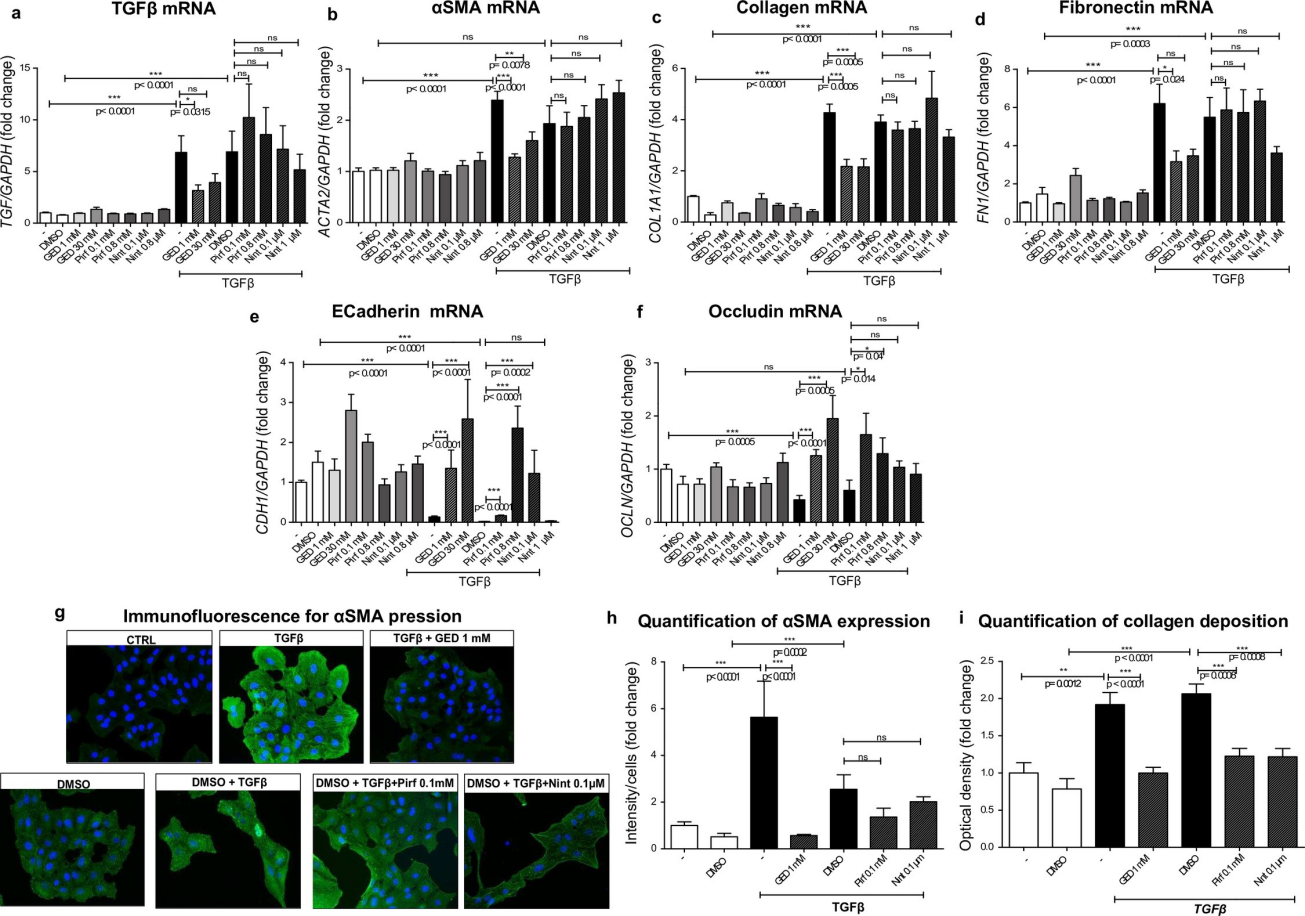
*In vitro* evaluation of the EMT in A549 cells. (a) Experimental scheme. The EMT was induced in human alveolar epithelial cells (A549) stimulated with 20 ng/mL TGFβ over four days. GED-0507 (1–30 mM), Pirf (0.1–0.8 mM) or Nint (0.1–1 μM) were administered at the same time of TGFβ stimulation. mRNA expression levels for mesenchymal cell markers (a) *TGFB1*, (b) *ACTA*, (c) *COL1A1*, (d) *FN1* and epithelial cell markers (e) *CDH1* and (f) *OCLN* were quantified in RT-PCRs and converted into histograms in 3 independent experiments performed in triplicate. (g) Microphotographs of immunofluorescence staining of α-SMA (green) were scanned at a magnification of 40x. Nuclei were counterstained using DAPI (blue). Digital images were processed with the Zeiss LSM Browser and (h) α-SMA protein expression was quantified on 3 microscope fields, using the threshold detection method with Image J software and represented on relative scatter plots. (i) Collagen deposition was quantified by picrosirius red staining and converted into histograms in 3 independent experiments performed in triplicate. Since Pirf and Nint were solubilized in DMSO, DMSO- and/or TGFβ+DMSO-treated cells were used as comparators in the statistical analysis. Data are quoted as the mean ± SEM fold change vs. CTRL cells. * = p< 0.05; ** = p< 0.01; *** = p< 0.001.

We next evaluated the expression of epithelial fibrosis markers in a monolayer of A549 cells (Fig 5). Four days of treatment with 20 ng/mL TGFβ over four days was associated with an increase in mRNA expression of *TGFB1*, *ACTA2*, *COL1A1* and *FN1*, and a decrease in mRNA expression of the genes coding for E-cadherin (*CDH1*) and occludin (*OCLN*). GED-0507 was able to restore the expression of all these genes to control levels, whereas Pirf and Nint reduced the overexpression of *TGFB* only (Fig 5A–5F). With regard to epithelial cell markers, Pirf (but not Nint) was able to restore mRNA levels of E-cadherin and occludin (Fig 5E and 5F).

Protein levels of α-SMA were significantly elevated in TGF-β-treated A549 cells. Collagen production (as measured by the optical density of the picrosirius red staining) was also enhanced. All three tested compounds were able to downregulate the TGF-β-induced post-transcriptional upregulation of α-SMA and collagen (Fig 5G and 5I). However, at both concentrations tested, the effect of GED-0507 was significantly greater than that of Pirf or Nint.

#### Evaluation of the ability of GED, Nint and Pirf to counter TGFβ-induced myofibroblast activation in primary HLFs

**Fig 6:**
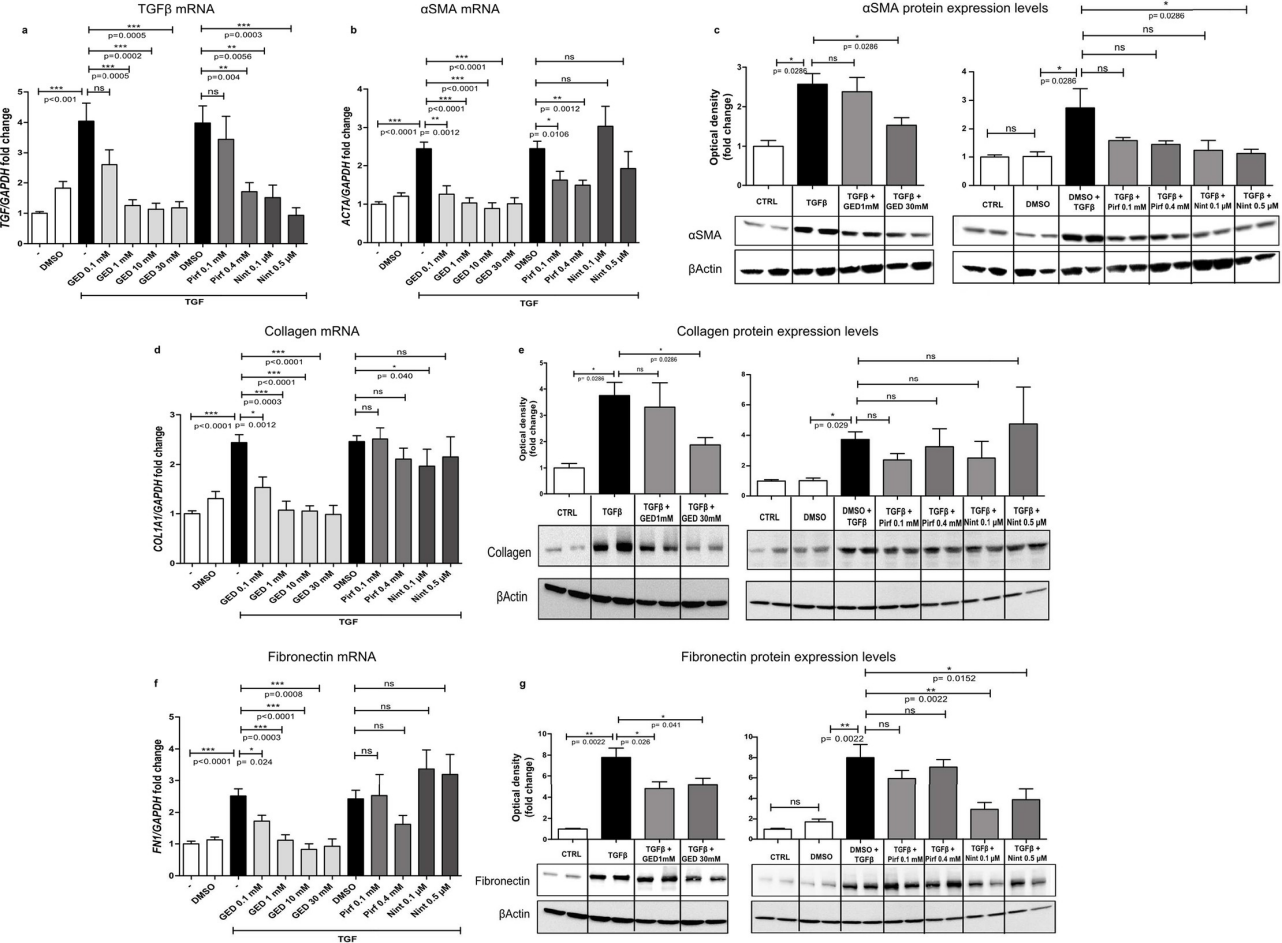
*In vitro* evaluation of myofibroblast activation in HLFs. Experimental plan. Myofibroblast activation was induced in primary human fibroblasts (HLFs) stimulated with 5 ng/mL TGFβ over two days. GED-0507 (1–30 mM), Pirf (0.1–0.4 mM) or Nint (0.1–0.5 μM) were administered at two different doses at the same time of TGFβ. mRNA expression levels for (a) *TGFB1*, (b) *ACTA*, (d) *COL1A1* and (f) *FN1* were quantified in RT-PCRs and converted into histograms for 3 independent experiments performed in triplicate. Protein expression levels for (c) α-SMA, (e) collagen and (g) fibronectin were evaluated on Western blots. Band density was quantified using ImageJ software and converted into histograms for 3 independent experiments in duplicate. Since Pirf and Nint were solubilized in DMSO, DMSO- and/or TGFβ+DMSO-treated cells were used as comparators in the statistical analysis. Data are quoted as the mean ± SEM fold change vs. CTRL cells. * = p< 0.05; ** = p< 0.01; *** = p< 0.001.

The fibroblast-to-myofibroblast transition in HLFs was induced by stimulation with 5 ng/mL TGFβ over two days (Fig 6). Starting at a concentration of 1 mM, all doses of GED-0507 were able to significantly decrease the expression of *TGFB1*, *ACTA2*, *COL1A1* and *FN1* genes, when compared with control TGFβ-stimulated cells. Pirf exerted only a limited inhibitory effect on *TGFB1* and *ACTA* genes, whereas Nint decreased *TGFB1* and *COL1A1* gene expression significantly when compared with TGFβ+DMSO-stimulated HLFs (Fig 6A, 6B, 6D and 6F). In Western blots, TGFβ-induced protein expression of α-SMA, collagen and fibronectin was also significantly reduced by 30 mM GED-0507. In contrast, Pirf was not able to reduce the post-transcriptional expression of all fibrosis markers, whereas Nint only reduced α-SMA and fibronectin protein levels at a higher concentration (0.5 μM) (Fig 6C, 6E and 6G).

## Discussion

As seen in other organs, fibrosis of the lung is characterized by the excessive deposition of ECM components. When this deposition is extensive, it can lead to parenchymal distortion and irreversible loss of function [26]. IPF is the prototypical, progressive, fibrotic lung disease, with a dismal prognosis and no cure. Although evidence-based guidelines on the treatment of IPF recommend the use of Pirf and Nint, these drugs only reduce the rate of functional decline or disease progression, and are associated with tolerability issues [27–29].

The TGFβ/SMAD pathway has a central role in the differentiation of fibroblasts to myofibroblasts, persistent myofibroblast activation, and aberrant ECM deposition that result in tissue fibrosis [30]. However, owing to TGFβ‘s pleiotropic effects, abrogation of the TGFβ pathway may not be an ideal strategy. Instead, it might be more effective to focus on pathways directly associated with TGFβ in either an agonistic or antagonistic context (such as IL-13 or PPAR-γ, respectively). The nuclear receptor PPAR-γ is naturally activated by prostaglandins (PG)-D1 and PG-D2) and the PG derivative 15-deoxy-Δ12 and 15d-PGJ2, and a target of several synthetic ligands. PPARγ is expressed in a wide range of tissues, including colon, spleen, retina, skeletal muscle, liver and lung. A better understanding of the many biological effects of ligand-dependent PPAR-γ activation has led to the widespread clinical use of synthetic PPAR-γ ligands, such as thiazolidinediones (TZDs) [31]. The ability of TZDs (especially rosiglitazone) to regulate the production of several inflammatory mediators (e.g., TNFα, IL-6, MCP1, and PAI-1) in a number of pathological conditions suggests that PPAR-γ exerts similar anti-inflammatory effects. Natural PPAR-γ ligands (e.g. 15d-PGJ2) and synthetic PPAR-γ ligands (e.g. rosiglitazone) also have antifibrotic effects [32]. Burgess et al. have shown that PPAR-γ agonists are able to modulate the differentiation of human lung primary fibroblasts into myofibroblasts following the addition of exogenous TGFβ [33]. PPAR-γ ligands (mainly the TZDs) are also able to (i) facilitate tissue regeneration and the resolution of fibrosis in murine models of BLM-induced lung fibrosis [32, 34–36], and (ii) prevent radiation-induced pulmonary fibrosis [37]. The beneficial effect of PPARγ activation in controlling lung fibrosis may be due to the receptor’s broad cellular distribution within the lung. Indeed, PPAR-γ are expressed by alveolar epithelial cells, fibroblasts, bronchial smooth muscle cells, type II pneumocytes, macrophages, endothelial cells, lymphocytes, and dendritic cells. PPAR-γ‘s functions depending on the cell type, which thus contributes to tissue homeostasis [38]. Conversely, the mechanisms underlying the antifibrotic properties of PPAR-γ activation in the lung remain to be elucidated. Despite their widespread use, full PPAR-γ agonists (such as the TZDs) are nevertheless associated with potentially serious adverse events [39]. However, PPAR-γ in the lung can be activated by a wide spectrum of compounds; these include SPPARMs, which induce specific conformational changes and thus resulting in differential activation and interactions with cofactors and corepressors. This strategy can avoid the adverse events associated with full agonists.

GED-0507 is a new SPPARM that binds with high affinity to the receptor’s ligand binding domain, as predicted by docking simulations [4]. We have previously shown that GED-0507 exhibits potent anti-inflammatory and antifibrotic properties and has a good safety and tolerability profile, thanks to its limited interference with lipid and glucose metabolism [2, 40, 41]. The latter effect has been demonstrated in studies of animals receiving very high oral doses of GED-0507 (including chronic toxicity studies in rats and in dogs, in which high systemic exposure to GED-0507 was achieved), and in Phase I and Phase II clinical studies of patients with inflammatory bowel disease [28]. GED-0507 also displays good absorption in the small intestine and significant retention in the lung. With regard to its biological activities, GED-0507 increases the expression of IkBα and thus preventing the nuclear translocation of NF-kB via transrepression [41]. Furthermore, we previously showed that exposure to GED-0507 counters the expression of genes coding for major profibrotic mediators (i.e., IL13 and the TGFβ/SMAD3 pathway), α-SMA and ECM components in mice with chronic intestinal inflammation [3]. In this context, GED-0507 appears to exert its antifibrotic effects through several mechanisms, including PPAR-γ activation and thus the maintenance of intestinal epithelial homeostasis and modulation of the epithelia-to-mesenchymal transition and the EMT [24, 42, 43].

The present study follows up on earlier work in which we showed that prophylactic oral administration of GED-0507 at the optimal dose of 100 mg/kg/day abolished the excessive mortality and weight loss observed three weeks after BLM administration in non-treated animals (24). When given with curative intent, GED-0507 reduced the mortality rate among mice receiving BLM by 50% and was associated with a weight gain similar to that seen in PBS-treated control animals without lung fibrosis. In BLM-treated mice, macroscopic lung lesions and specific quantitative readouts of lung fibrosis (such as the hydroxyproline content and Ashcroft’s score) were also reduced by 57% and 47%, respectively [24].

A gain-of-function polymorphism within the promoter region of the *MUC5B* gene (rs35705950) is the strongest genetic risk factor for both familial and sporadic forms of IPF [44]). MUC5B is the main secretory mucin in the superficial epithelium and glands, and its expression is regulated by NF-κB and activating protein-1 [45, 46]. In patients with IPF, overexpression of MUC5B in the distal airways is believed to impair mucociliary clearance and thus lead to excessive lung retention of xenobiotics or endogenous inflammatory debris, which may induce and sustain the development of fibrosis [47]. Notably, GED-0507 was able to restore MUC5B overexpression in the lung of mice receiving BLM.

The main objective of our study was to demonstrate GED-0507’s antifibrotic effects *in vitro* on lung epithelial cells and fibroblasts and *in vivo* in a mouse model of experimentally-induced pulmonary fibrosis. We also compared GED-0507 with Pirf and Nint, the two drugs approved for the treatment of IPF. Our choice of the dose levels of GED-0507, Pirf and Nint used here was based on (i) a dose-response evaluation in A549/MRC-5 cell lines and primary HLFs, (ii) *in vivo* data in medical databases on the optimal dosages of Pirf and Nint in BLM-induced lung fibrosis [48–53], and (iii) the dose level of 100 mg/Kg for GED-0507 used in toxicological studies of animals. Chronic administration of increasing concentrations of GED-0507 for 26 days in rats enabled definition of the “no observed adverse effect level” (NOAEL) as 500 mg/Kg/day [54]. In line with the FDA’s draft guidelines on dose conversion between animals and humans, the human equivalent dose for GED-0507 is 81 mg/kg/day (based on body surface area), with a maximum human recommended starting dose of 8 mg/kg (the human equivalent dose divided by a safety factor of 10). In a phase IIb clinical trial in inflammatory bowel disease, GED was administered at a dose of 8 mg/kg/day; with a conversion factor of 0.08, this corresponds to 100 mg/Kg/day in the mouse. Based on the same conversion criteria, we administered Pirf and Nint at doses of 400 mg/Kg/day and 60 mg/kg/day, respectively. These are the most commonly used doses in murine models of lung fibrosis and correspond to the US Food and Drug Administration’s recommended daily dose for the treatment of patients with IPF (2403 mg/d for Pirf and 300 mg/d for Nint) [55]. When evaluated under the same conditions, GED-0507 displayed more pronounced antifibrotic activity *in vitro* and *in vivo* than Pirf and Nint for most biomarkers associated with alveolar epithelial cell dysfunction, the EMT, and ECM remodeling.

Our study had several limitations. Firstly, we used A549 cells. Although a number of studies have reported on the use of A549 cells as a model of human alveolar epithelial cells [56], GED-0507’s antifibrotic properties need to be confirmed in other cell types, such as alveolar epithelial cells or small airway epithelial cells. Secondly, since one of the features of the BLM model is loss of epithelium, it may be difficult to distinguish between the EMT and loss of epithelium. However, we observed a clear fibrotic profile 28 days after BLM administration. In this regard, a number of studies in mice have shown that BLM administration can induce the EMT [57–59]. Lastly, the mechanisms by which GED-0507-induced modulation of PPAR-γ signaling attenuates lung fibrosis remain to be elucidated.

In conclusion, we provided further evidence of the antifibrotic effects of GED-0507 by demonstrating the compound’s ability to modulate several fibrosis pathways in BLM-induced pulmonary fibrosis and in TGFβ-exposed lung epithelial cells and fibroblasts.

GED-0507 has displayed a very favorable safety and tolerability profile in both preclinical and clinical studies. The compound’s preclinical safety profile derives from pharmacology studies in rats and dogs, chronic toxicity studies (with up to 26 weeks of repeated-dose oral administration) in rats and 39 weeks in dogs), embryo-fetal development toxicity studies in rats and rabbits, and genotoxicity studies. No effects were observed on either cardiovascular parameters in dogs or nervous and respiratory function in rats at doses up to 2000 mg/kg (the NOAEL). GED-0507 has also proven to be safe and well tolerated in patients with ulcerative colitis at doses of 160 mg/day and 320 mg/day for 8 consecutive weeks. Specifically, no clinically meaningful abnormalities in laboratory tests (hematology, biochemistry, and urine analyses), vital signs, physical findings, or the ECG occurred in any of the subjects during the study period.

GED-0507’s potential mechanisms of action of GED-0507 are represented in S3 Fig. GED-0507 is now ready to enter a Phase Ib trial in patients with IPF.

## Supporting information

S1 Fig Outline of experimental design for (a) preventive and (b) curative treatments.
(TIF)

S2 Fig GED does not show cytotoxic effects in A549 and MRC-5 cell lines and in HLF. Cell viability assay in response to growing concentrations of GED, Pirf and Nint in (a) A549, (b) MRC-5 and (c) HLF cells. Data are expressed as mean of fold changes ± SEM; * = p< 0.05; ** = p< 0.01; *** = p< 0.001.
(TIF)

S3 Fig Schematic pathophysiology of pulmonary fibrosis. The onset of fibrotic process in lung begins from repeated injuries which lead to inflammatory response, via the activation of the NFκB pathway and consequent release of pro-inflammatory cytokines. Uncontrolled overproduction of further cytokines and growth factors, among which TGFβ, induces the succession of a series of reversible cellular processes which culminate in lung fibrosis. Those processes are schematically represented in the figure and include: death of Type I alveolar epithelial cell (AEC), proliferation of Type II alveolar epithelial cells which undergo the epithelial-mesenchymal transition (EMT) and a consequent failure of re-epithelialization, fibroblasts recruitment and proliferation, myofibroblasts activation, ECM deposition and MUC5B production.
(TIF)

S1 Table Mouse primer sequences for quantitative RT-PCR. (PDF)

S2 Table Human primer sequences for quantitative RT-PCR. (PDF)

S1 Data (PDF)

## Abbreviations

PPAR-γ: Peroxisome proliferator-activated receptor gamma
BLM: Bleomycin
TGFβ: Transforming growth factor beta
EMT: Epithelial-to-mesenchymal transition
SPPARM: Selective peroxisome proliferator-activated receptor modulator
DSS: Dextran Sulfate
α-SMA: alpha-smooth muscle actin
ECM: Extracellular matrix
IPF: Idiopathic pulmonary fibrosis
Pirf: Pirfenidone
Nint: Nintedanib
HLF: Human lung fibroblast
DMSO: Dimethyl sulfoxide
MUC5B: Mucin 5B
IL: Interleukin
TNF-α: Tumor necrosis factor alpha
*Tgfb/TGFB*: TGFβ1 gene
*Acta2/ACTA2*: α-SMA gene
*Col1A1/COL1A1*: Collagen type 1 α1 gene
*Fn1/FN1*: Fibronectin gene
*Cdh1/CDH1*: E-cadherin gene
*Ocln/OCLN*: Occludin
PG: Prostaglandin
TZD: Thiazolidinedione
