# Supplementary figures and images for: GED-0507 attenuates lung fibrosis by counteracting myofibroblast transdifferentiation in vivo and in vitro
Source: PLoS One. 2021 Sep 16;16(9):e0257281. doi: 10.1371/journal.pone.0257281 (PMC8445472; doi:10.1371/journal.pone.0257281)

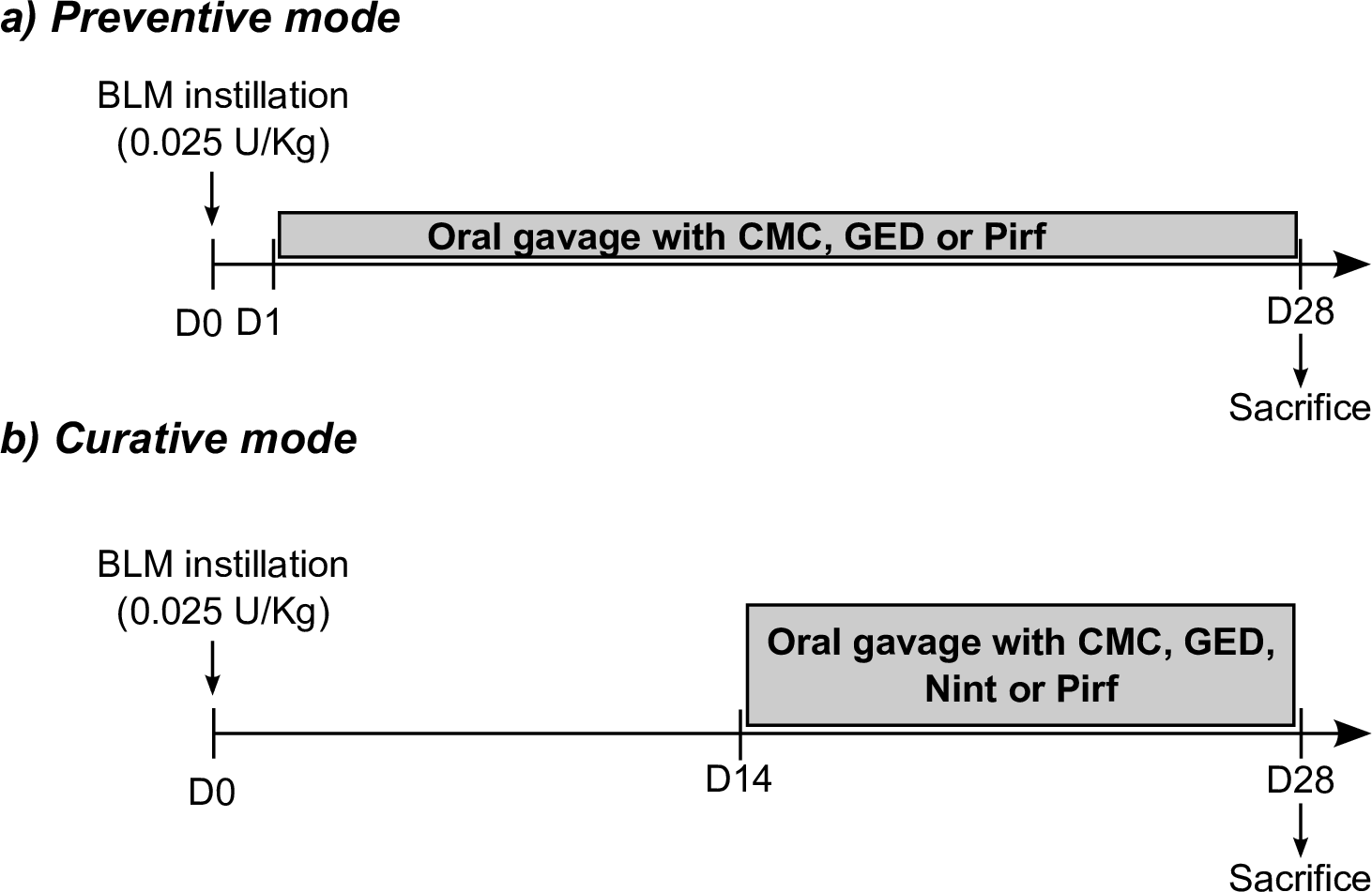

Supplement: S1 Fig — Outline of experimental design for (a) preventive and (b) curative treatments. (TIF) [file pone.0257281.s001.tif]

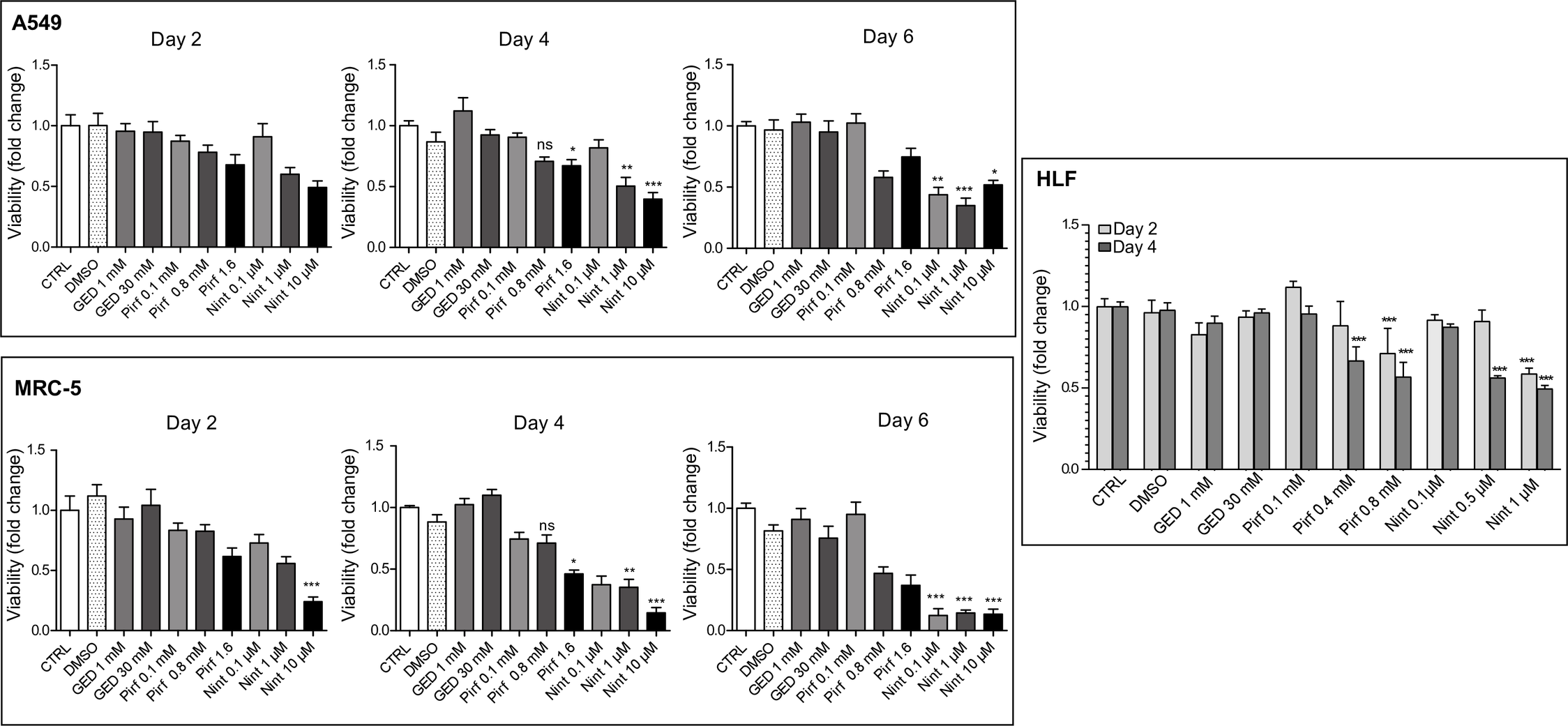

Supplement: S2 Fig — Cell viability assay in response to growing concentrations of GED, Pirf and Nint in (a) A549, (b) MRC-5 and (c) HLF cells. Data are expressed as mean of fold changes ± SEM; * = p< 0.05; ** = p< 0.01; *** = p< 0.001. (TIF) [file pone.0257281.s002.tif]

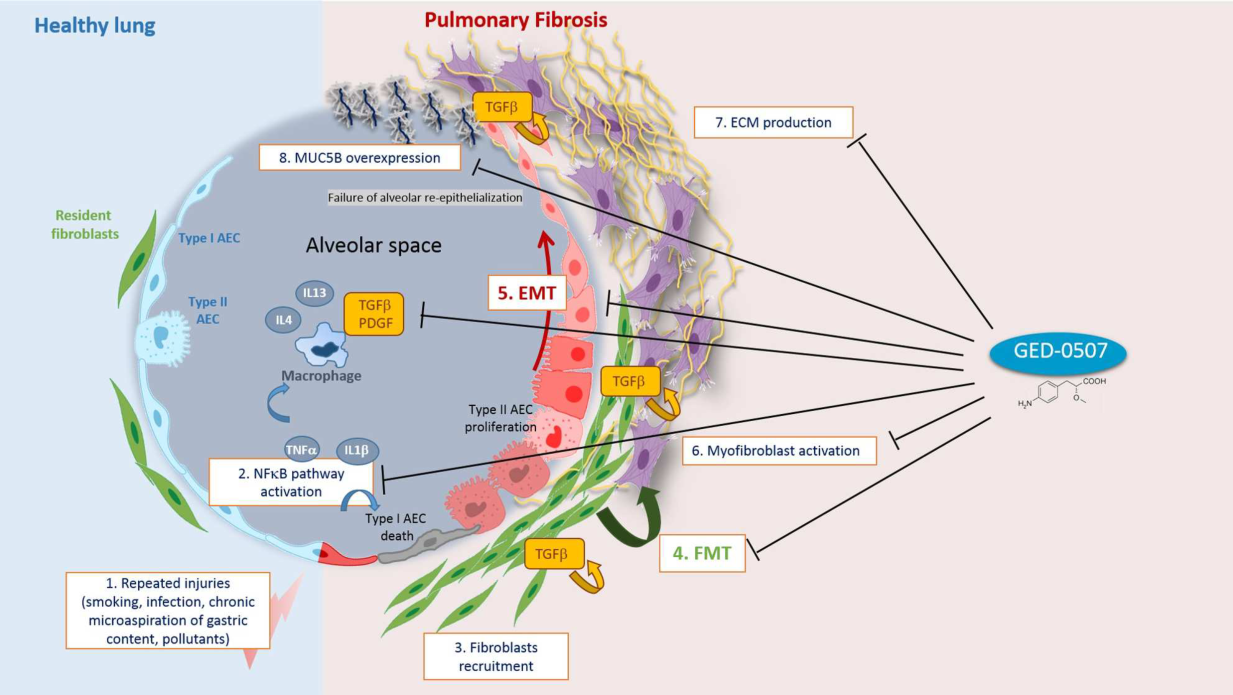

Supplement: S3 Fig — The onset of fibrotic process in lung begins from repeated injuries which lead to inflammatory response, via the activation of the NFκB pathway and consequent release of pro-inflammatory cytokines. Uncontrolled overproduction of further cytokines and growth factors, among which TGFβ, induces the succession of a series of reversible cellular processes which culminate in lung fibrosis. Those processes are schematically represented in the figure and include: death of Type I alveolar epithelial cell (AEC), proliferation of Type II alveolar epithelial cells which undergo the epithelial-mesenchymal transition (EMT) and a consequent failure of re-epithelialization, fibroblasts recruitment and proliferation, myofibroblasts activation, ECM deposition and MUC5B production. (TIF) [file pone.0257281.s003.tif]
