# Supplementary material for: GED-0507 attenuates lung fibrosis by counteracting myofibroblast transdifferentiation in vivo and in vitro
Source: PLoS One. 2021 Sep 16;16(9):e0257281. doi: 10.1371/journal.pone.0257281 (PMC8445472; doi:10.1371/journal.pone.0257281)
Supplement: S1 Table — (PDF) [file pone.0257281.s004.pdf]

Gene	Source	Primer	Sequences (5' → 3')
TGFβ (Tgfb1)	Mouse	Forward	CCCGAAGCGGACTACTATGCT
		Reverse	GTTTTCTCATAGATGGCGTTGTTG
αSMA (Acta2)	Mouse	Forward	CCT GAC GGG CAG GTG ATC
		Reverse	ATG AAA GAT GGC TGG AAG AGA GTC T
Collagen (Col1a1)	Mouse	Forward	GAG TAC TGG ATC GAC CCT AAC CAA
		Reverse	ACA CAG GTC TGA CCT GTC TCC AT
Fibronectin (Fn1)	Mouse	Forward	CGAAGCCGGGAAGAGCAAG
		Reverse	CGTTCCCACTGCTGATTTATCTG
GAPDH (Gapdh)	Mouse	Forward	ATG GGA AGC TTG TCA TCA ACG
		Reverse	GGC AGT GAT GGC ATG GAC TG
IL-1β (Il-1b)	Mouse	Forward	CAACCAACAAGTGATATTCTCCATG
		Reverse	GATCCACACTCTCCAGCTGCA
TNFα (Tnf)	Mouse	Forward	CCACCACGCTCTTCTGTCTA
		Reverse	GAGGCCATTTGGGAACTTCT
MUC5B (Muc5b)	Mouse	Forward	GCACGTAAATGCGACTGTCT
		Reverse	ATGGACCTTGCTCTCCTGAC
E-Cadherin (Cdh1)	Mouse	Forward	CACCTGGAGAGAGGCCATGT
		Reverse	TGGGAAACATGAGCAGCTCT
Occludin (Ocln)	Mouse	Forward	CCCTGACCACTATGAAACAG
		Reverse	TTGATCTGAAGTGATAGGTG
