# Supplementary material for: GED-0507 attenuates lung fibrosis by counteracting myofibroblast transdifferentiation in vivo and in vitro
Source: PLoS One. 2021 Sep 16;16(9):e0257281. doi: 10.1371/journal.pone.0257281 (PMC8445472; doi:10.1371/journal.pone.0257281)
Supplement: S2 Table — (PDF) [file pone.0257281.s005.pdf]

Gene	Source	Primer	Sequences (5' → 3')
αSMA (ACTA)	Human	Forward	CCTTCCAGCAGATGTGGATCA
		Reverse	AAGCATTTGCGGTGGACAA
Fibronectin (FN1)	Human	Forward	GATGCTCCCACTAACCTCCA
		Reverse	CGGTCAGTCGGTATCCTGTT
Collagen (COL1A1)	Human	Forward	TGGGCGGGAGAGACTGTTC
		Reverse	TGCCCCGGTGACACATC
E-Cadherin (CDH1)	Human	Forward	CAACTATAGCGAGCTGCAGGA
		Reverse	ACTTGGGGTACCAGGAGAGAGT
Occludin (OCLN)	Human	Forward	AGGACGTGCCTTCACCCCCA
		Reverse	ACCACCGCTGCTGTAACGAGG
TGFβ (TGFB1)	Human	Forward	AAC-CCA-CAA-CGA-AAT-CTA-TGA-CAA-G
		Reverse	AGA-GCA-ACA-CGG-GTT-CAG-GTA
GAPDH (GAPDH)	Human	Forward	GAC-ACC-CAC-TCC-TCC-ACC-TTT
		Reverse	TTG-CTG-TAG-CCA-AAT-TCG-TTG-T
