# Supplementary material for: GED-0507 attenuates lung fibrosis by counteracting myofibroblast transdifferentiation in vivo and in vitro
Source: PLoS One. 2021 Sep 16;16(9):e0257281. doi: 10.1371/journal.pone.0257281 (PMC8445472; doi:10.1371/journal.pone.0257281)

Original Blot Fig 6c: βActin

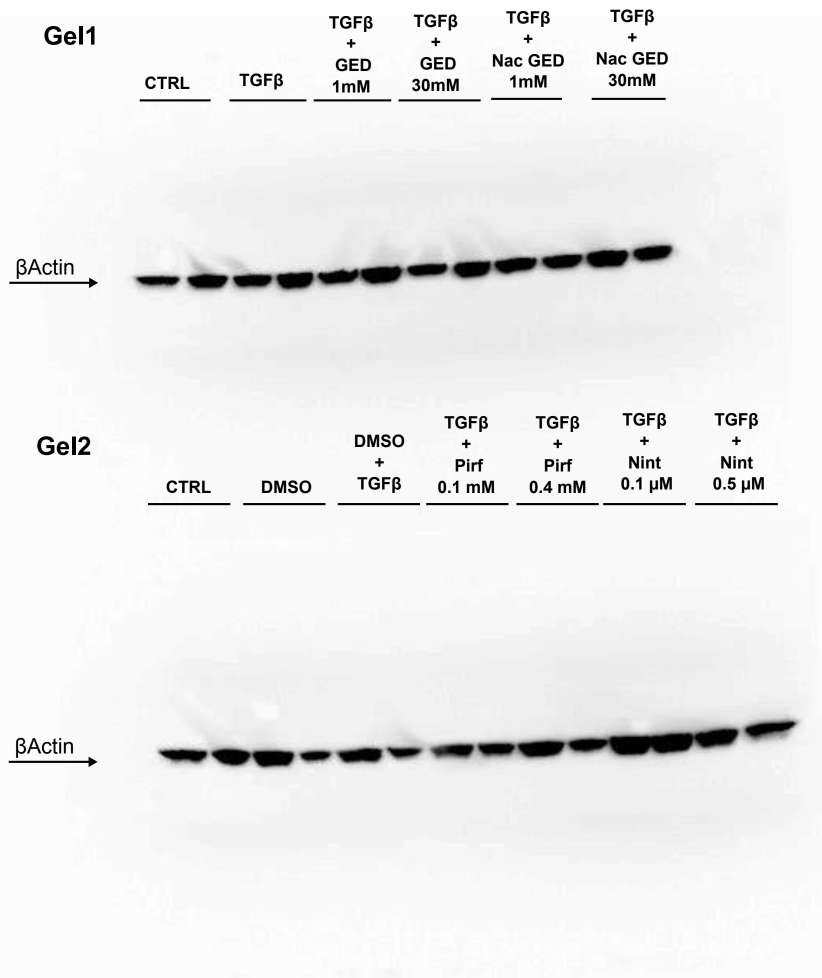

Original Blot Fig 6 and f: βActin

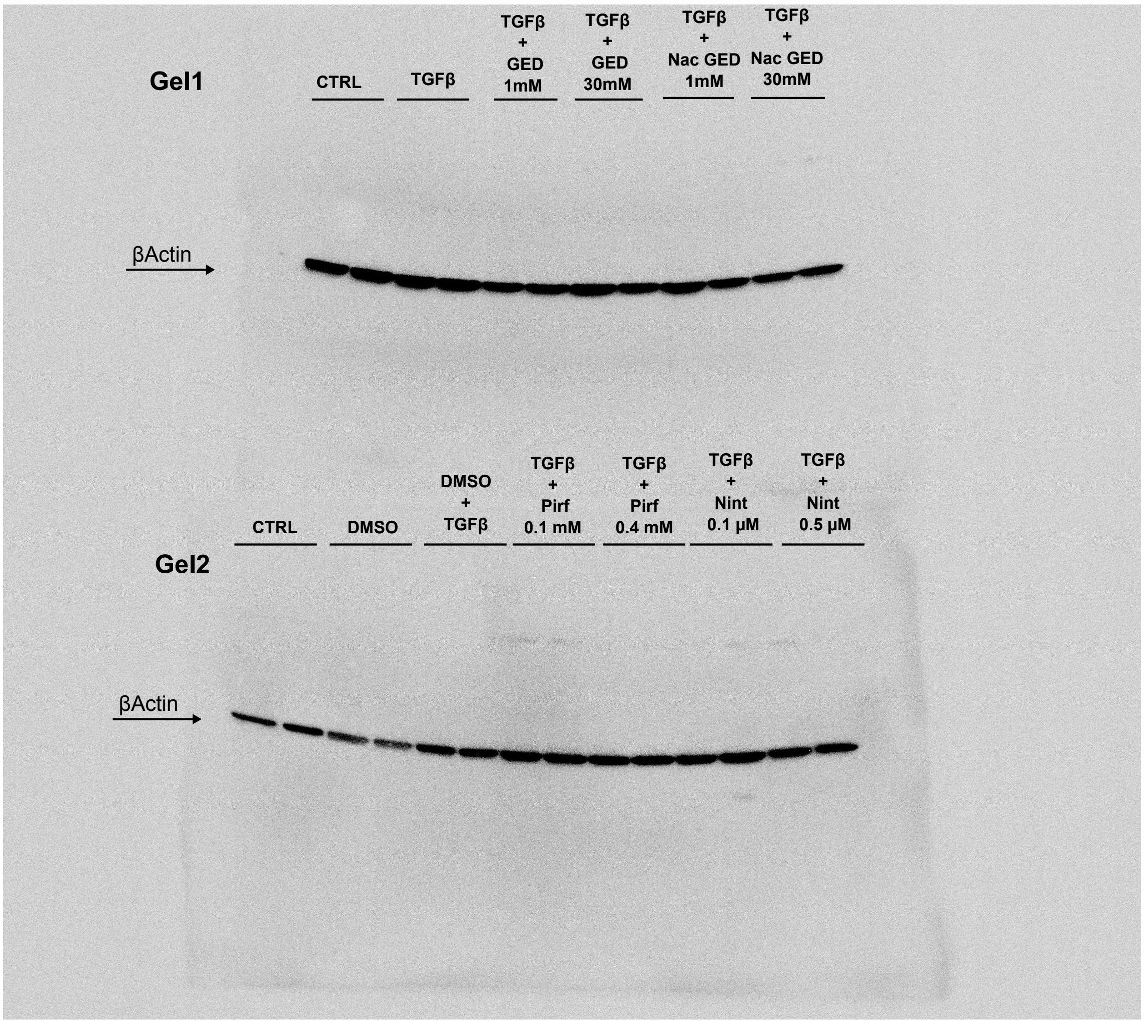

Original Blot Fig 6e: Collagen

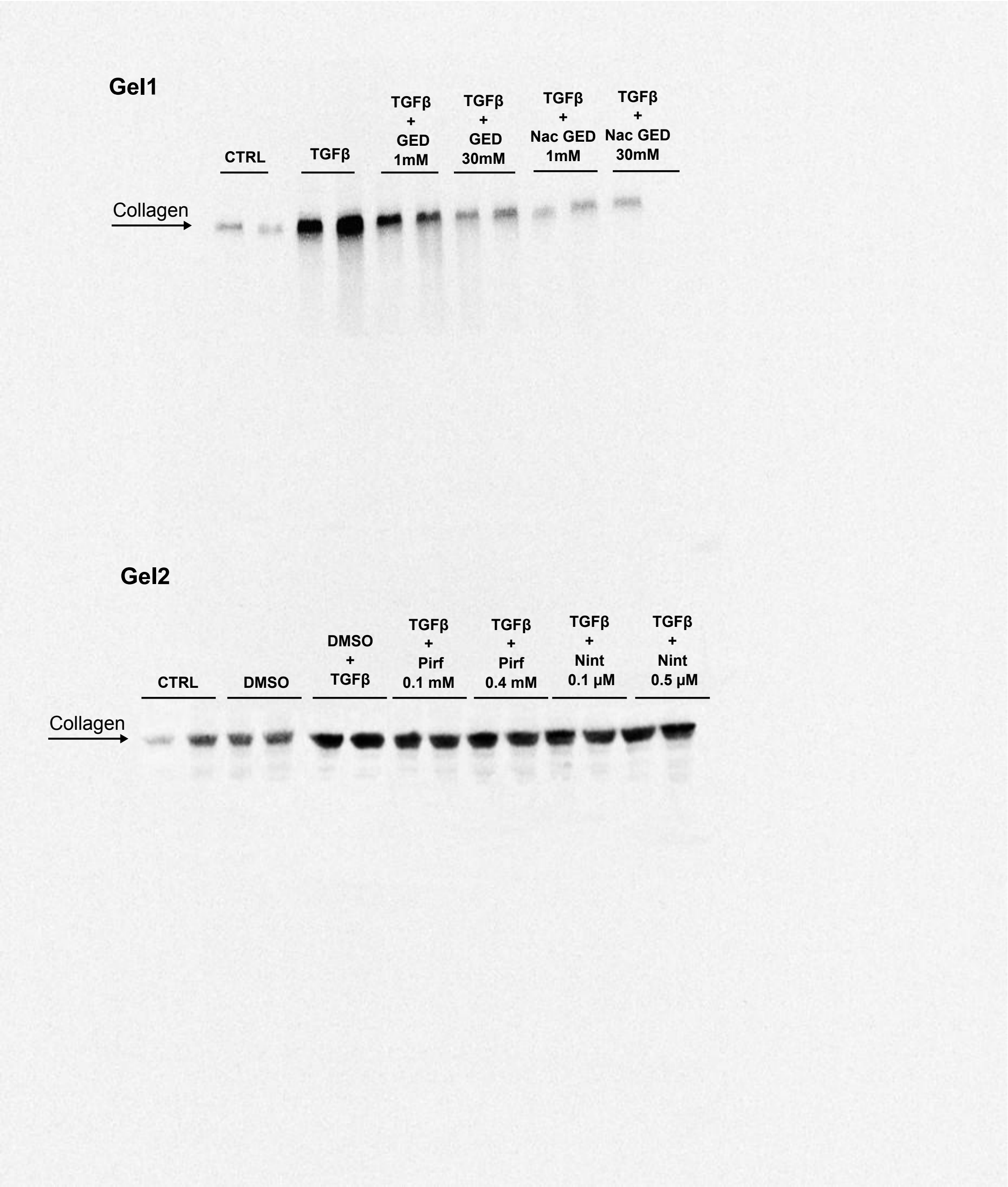

Original Blot Fig 6g: Fibronectin

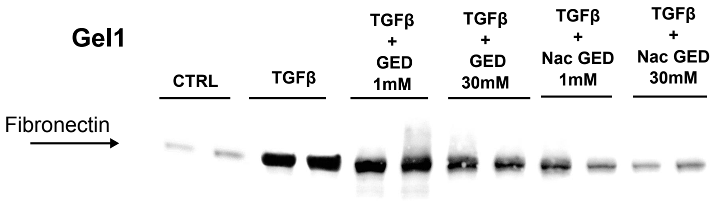

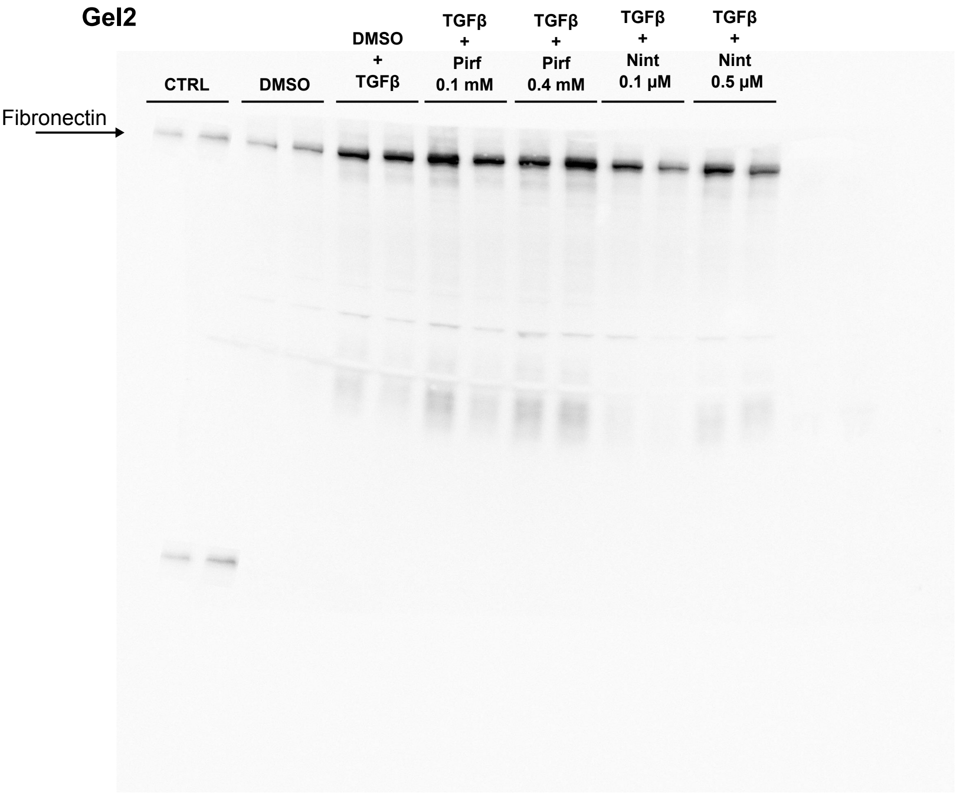

Supplement: S1 Data — (PDF) [file pone.0257281.s006.pdf]
